# Foam Formation in Shake Flasks and Its Consequences

**DOI:** 10.1002/elsc.70057

**Published:** 2025-10-22

**Authors:** David Vonester, Kyra Hoffmann, Thomas Palmen, Lena Regestein, Ulrike Richter, Anna‐Lena Altenhoff, Maximilian Hoffmann, Yulia Radeva, Jochen Büchs, Jørgen Barsett Magnus

**Affiliations:** ^1^ AVT – Biochemical Engineering, RWTH Aachen University Aachen Germany

**Keywords:** foam formation, maximum oxygen transfer capacity, online viscosity measurement, out‐of‐phase phenomenon, shake flask

## Abstract

Foam formation in stirred tank fermentation processes is a well‐studied phenomenon. However, foaming in shake flask cultivations is rarely considered. Non‐baffled shake flasks, in particular, are generally considered to prevent foaming problems. However, under certain process conditions, foaming in non‐baffled shake flasks can occur. In this study, phenomena of foam formation in shake flasks, their impact on the maximum oxygen transfer capacity (OTR_max_), and experimental reproducibility are investigated. It is shown that foaming events in shake flasks can increase the OTR_max_ by up to threefold. This enhanced OTR_max_ alters process conditions and, thereby, affects the reproducibility of experiments. Foaming in shake flasks can be induced by elements such as conventional baffles or sensor spots that are used for online measurement. Moreover, a connection between the out‐of‐phase phenomenon and foam formation was discovered in non‐baffled shake flasks. This is especially important when cultivating microorganisms at elevated viscosities. Hence, foaming in shake flasks should be considered as significantly altering process conditions, compared to non‐foaming cultures. Ensuring in‐phase cultivation conditions and unhindered liquid flow in shake flasks may help to avoid foaming.

*Practical application:* This work provides insights into foam formation in non‐baffled shake flasks and its resulting implications. Foaming can increase the maximum oxygen transfer capacity and, thus, affect process conditions. The reproducibility can be severely reduced, and a comparison between foaming and non‐foaming cultivations is only possible to a limited extent. Foaming can be induced by baffles or internals, such as small sensor spots, used for online monitoring. Additionally, foaming can be caused by the out‐of‐phase phenomenon. This is of particular importance when cultivating microorganisms at elevated viscosities. This paper is intended to raise awareness of the topic of foam formation in the shake flask and help to correctly interpret this phenomenon.

## 1 Introduction

Foam formation in biotechnological processes is a widespread phenomenon. It is commonly associated with stirred tank reactors, although foam formation is also observed in shake flask cultivations. Foam is described as an accumulation of gas bubbles finely dispersed in a liquid [1]. The volume fraction of gas in liquid usually has a share of 50–97 vol.% [2]. The liquid fraction of the foam directly affects its complex structure, and it varies due to liquid drainage caused by gravitational forces. Thus, the liquid amount at the top of a foam layer is the lowest, increasing from top to bottom. Foam with a gas fraction of more than 95 vol.% is described as dry foam, or polyhedral foam [3]. In contrast, foam with a gas fraction of more than 85 vol.% is described as wet foam, or spherical foam [3]. The gas‐filled cavities of dry, polyhedral foam have a characteristic angular shape. The intersections between two gas compartments are called membranes or lamellas and are aligned at a certain angle to each other. When three lamellas meet, the angle is 120°, when four lamellas meet, the angle is 109.5° [4]. Considering wet foam, the gas‐filled cavities have a spherical structure [5]. The ability of foam formation is controlled by liquid properties like viscosity, surface tension, or ionic strength [6]. In fermentation processes, these parameters are affected by the medium compositions, the microorganisms themselves, metabolites, or residues of the microorganisms, such as cell membranes after cell lysis [6, 7]. Amphiphilic or hydrophobic molecules migrate to the lamella between the gas and liquid phase and, thus, stabilize the foam [7]. In biotechnological processes, foam formation is promoted by heavy agitation of the fermentation broth and intense gassing. Excessive foam formation can have severe impacts on the fermentation process. It might occupy large fractions of the fermentation vessel and might be blown in the exhaust system and clog the exhaust filter or lead to a malfunction of probes or sensors [1]. Moreover, foam might affect the composition of the fermentation medium by the removal of medium components or microorganisms from the liquid phase and accumulation in the foam [8, 9]. The entrainment of medium components, such as products, might be a beneficial effect and is utilized by the foam fractionation process [8, 10]. Foaming can be suppressed by mechanical measures, such as physical foam breakers, or chemicals such as antifoam agents. However, antifoam agents can affect the oxygen transfer into the liquid phase by increasing the coalescence behavior of air bubbles in the fermentation broth. Furthermore, antifoam agents can also complicate downstream processing, as they usually act as surfactants that interfere with separation processes (e.g., membrane filters) [7]. On a smaller scale, in shake flasks, foaming is usually less of a problem. This is due to the surface aeration, in contrast to submerged bubble aeration in stirred tank reactors [11]. However, when working with baffled shake flasks, foaming is also frequently observed [12, 13, 14, 15]. Baffled shake flasks are utilized due to the higher power consumption, leading to a higher maximum oxygen transfer capacity (OTR_max_). The baffles cause vigorous mixing and splashing of the fermentation broth, which often leads to foaming. Therefore, baffled shake flasks must be treated with caution when dealing with bioprocesses tending to foaming. In contrast, non‐baffled shake flasks are, in general, considered to prevent foaming [16]. This property is particularly beneficial for the production of surface‐active substances, such as surfactin [17]. It can be impossible to prevent foaming when producing surfactin by *Bacillus subtilis* in a stirred tank reactor. In contrast, foaming does not occur in non‐baffled shake flasks [18]. Although non‐baffled shake flasks are considered to avoid foaming, there are a few examples in literature that nonetheless describe foam formation in non‐baffled shake flasks. However, the cause of these foaming events remains partly unexplained [19, 20].

This study aims to elucidate the principles and effects of foam formation in shake flasks, focusing on non‐baffled shake flasks. This topic has rarely been dealt with, although foaming in shake flasks can have severe impacts on the process characteristics. The effects of foaming on the oxygen transfer and the reproducibility of cultivations in shake flasks are disclosed. Moreover, conditions leading to foam formation in non‐baffled shake flasks are presented and analyzed.

## 2 Materials and Methods

### 2.1 Sulfite System

For measurements of the OTR_max_, caused by different types of baffles, a 1 M sulfite system was prepared according to Meier et al. [21]. The experiments were conducted in 500 mL shake flasks with a filling volume of 30 mL, a shaking frequency of 150 rpm, and a shaking diameter of 50 mm at a temperature of 22.5°C. To investigate the influence of foam on the OTR_max_, 0.3 vol.% Tween 20 (Carl Roth GmbH, Karlsruhe, Germany) was added to the sulfite system.

### 2.2 Microorganisms and Culture Media

Precultures and main‐cultures of *Escherichia coli* VH33 were conducted in terrific broth (TB) medium, prepared according to Losen et al. [22] (see Table S1). For main‐cultures, 10 µL of Tween 20 per 100 mL of filling volume was added to the cultivation medium to increase the foaming tendency. Precultures were inoculated with 400 µL of cryogenic culture per 100 mL of cultivation broth. The cultivation was conducted in 250 mL shake flasks with a filling volume of 10 mL, a shaking frequency of 250 rpm, and a shaking diameter of 50 mm at a temperature of 37°C. The cultivation time was 3 h. Main‐cultures were inoculated with 400 µL of preculture per 100 mL of cultivation broth. The cultivation was conducted in 500 mL RAMOS flasks with a filling volume of 100 mL, a shaking frequency of 250 rpm, and a shaking diameter of 50 mm at a temperature of 37°C. Two out of four RAMOS flasks contained one commercially available pH sensor spot each (PreSens Precision Sensing GmbH, Regensburg, Germany), aseptically glued with silicone glue to the inner glass wall at the height of the largest inner flask diameter.

For the cultivation of *Bacillus subtilis* Pxyl Δspo, a two‐stage preculture was performed. For the first preculture, a cryogenic culture was inoculated into lysogeny broth (LB) medium, prepared according to Hoffmann et al. [23] (see Table S2). All cultivations of *B. subtilis* were conducted in 250 mL shake flasks with a filling volume of 20 mL, a shaking frequency of 350 rpm, and a shaking diameter of 50 mm at a temperature of 37°C. For the second preculture, modified V3 glucose minimal medium, prepared according to Hoffmann et al. [23] (see Table S3), was inoculated to an initial optical density (OD_600_) of 0.1 from the first preculture. The main‐culture was also conducted in modified V3 glucose minimal medium with 1 g/L xylose for the induction of poly‐γ‐glutamic‐acid (γ‐PGA) production [24]. A master mix with an initial optical density (OD_600_) of 0.1 with cells from the second preculture was prepared and distributed between the shake flasks. The shake flasks for offline sampling and the RAMOS flasks for online monitoring were cultivated under identical conditions to ensure comparability.

Precultures and main‐cultures of *Paenibacillus polymyxa* DSM 365 were performed in MM1P100 medium. The medium was prepared according to Sieben [25] (see Tables S4–S5) and contained 17.5 g/L glucose for preculture and 30 g/L glucose for main‐culture. Precultures were inoculated from a cryogenic culture to an initial optical density (OD_600_) of 0.1. The cultivation was conducted in 250 mL shake flasks with a filling volume of 30 mL, a shaking frequency of 300 rpm, and a shaking diameter of 50 mm at a temperature of 30°C. The cultivation time was approximately 9 h, and the cells were harvested in the late exponential phase. Shake flasks for offline sampling and RAMOS flasks for online monitoring were inoculated prior to the experiment to an initial optical density (OD_600_) of 0.5. The main‐culture was performed under the same cultivation conditions as the preculture, but at a shaking frequency of 200 rpm. For online measurement of the viscosity, 0.2 mg/mL fluorescent Oxnano nanoparticles (PyroScience GmbH, Aachen, Germany) were added to the cultivation broth. The shake flasks for offline sampling and the RAMOS flasks for online monitoring were cultivated under identical conditions to ensure comparability.

### 2.3 Online Monitoring

The oxygen transfer rate (OTR) of the cultivations was online monitored with a RAMOS (Respiration Activity Monitoring System) device. The measurement principle of the RAMOS technology is described in detail elsewhere [26, 27]. Online monitoring of the viscosity of *B. subtilis* was performed with a ViMOS (Viscosity Monitoring Online System) device. The measurement principle is described in detail by Sieben et al. [28]. In up to eight shake flasks, the shift of the angular position of the rotating bulk liquid *θ–θ*_0_ is measured relative to the direction of the centrifugal acceleration of the lab shaker. The measurement is performed non‐invasively by transmitted light measurements at a wavelength of 950 nm. A calibration, using standards with known viscosity, enables the online measurement of the viscosity during cultivation. For online monitoring of the viscosity of *P. polymyxa*, a ShakeVisc device was used. The measurement principle is described in detail by Dinter et al. [19]. Similar to the ViMOS technology, presented by Sieben et al. [28], the shift of the angular position of the rotating bulk liquid *θ–θ*_0_ is measured relative to the centrifugal acceleration of the lab shaker. However, in contrast to the ViMOS technology, the measurement is performed by back‐scattered fluorescence measurements at an excitation wavelength of 610–630 nm and an emission wavelength of 760–790 nm. Therefore, fluorescent Oxnano nanoparticles are added to the cultivation broth. The shift of the angular position of the rotating bulk liquid *θ–θ*_0_ correlates to the offline measured viscosity. A calibration of the ShakeVisc device is also possible. For online monitoring of biomass, back‐scattered light measurements at a wavelength of 610–630 nm were performed.

### 2.4 Offline Analysis

Shake flasks with paper plugs for offline sampling were cultivated under the same conditions as RAMOS flasks for online monitoring to ensure comparable conditions. The offline viscosity was determined with a rheometer (Physica MCR 301, Anton Paar GmbH, Osfildern‐Scharnhausen, Germany) at the respective cultivation temperature. If not otherwise stated, measurements were performed in triplicate. For measurements of cultivation broth of *B. subtilis*, the rheometer was equipped with a cone‐plate system (CP50‐0.5/TG, cone truncation 54 µm, cone angle 0.467°), and measurements were conducted at a shear rate of 100–5000 s^−1^. For measurements of cultivation broth of *P. polymyxa*, the rheometer was equipped with a cone‐plate system (CP50‐0.5/TG, cone truncation 56 µm, cone angle 0.475°) and measurements were conducted at a shear rate of 10–5000 s^−1^. Cultivation broths are commonly of non‐Newtonian behavior. The apparent viscosity is dependent on the average shear rate. To determine the offline viscosity with rheometer measurements, the shear rate in the shake flask at each individual sampling point has to be calculated. The shear rate that prevails in the shake flask can then be mimicked with the rheometer to derive the viscosity. The exact procedure is described by Giese et al. [29] and Sieben et al. [28]. The offline optical density was measured at a wavelength of 600 nm with a photometer (Genesys 20, Thermo Scientific, Dreieich, Germany). Samples were diluted with a 9 g/L NaCl solution to an optical density between 0.1 and 0.3. Measurements were performed in triplicate.

### 2.5 Theory of the Out‐of‐Phase Phenomenon

In this work, cultivations at elevated viscosities are presented. These cultivations in non‐baffled shake flasks can be affected by the out‐of‐phase phenomenon, which was first described by Büchs et al. [30, 31]. In out‐of‐phase operating conditions, the bulk liquid cannot follow the rotating centrifugal force of the orbital shaking motion of the lab shaker. The rotating bulk liquid collapses, resulting in an undefined, reduced liquid motion and, hence, a lowered power input, insufficient mixing, and reduced oxygen transfer. In contrast, in‐phase operation conditions describe the state, when the rotating bulk liquid follows the centrifugal force of the rotating lab shaker. To quantify the transition between in‐phase conditions to out‐of‐phase conditions, Büchs et al. developed the dimensionless phase number [31]. This phase number should be regarded as a parameter used for orientation, rather than as a fixed limit, since the transition to the out‐of‐phase flow behavior is inherently gradual and not sudden. Based on volumetric power input measurements, Büchs et al. derived a critical phase number of 1.26 [31]. Values below the critical phase number are considered out‐of‐phase. Later, Azizan et al. studied liquid distributions in shake flasks and reevaluated the phase number to 0.91 [32]. The definition of the Phase number, according to Büchs et al. [31], is shown in Equation (1):

(1) $$\mathrm{Ph}=\frac{d_{0}}{d}\cdot \left(1+3\cdot \log_{10}\left(Re_{\mathrm{film}}\right)\right)$$

Ph is the phase number, Re_film_ is the Reynolds film number, *d*_0_ is the shaking diameter, and *d* is the largest inner flask diameter. The Reynolds film number can be expressed as a function of the Reynolds number, as shown in Equation (2):

(2) Refilm=Re·π2·1−1−4π·VL13/d22

where Re is the Reynolds number and *V*_L_ is the filling volume. The definition of the Reynolds number Re for shake flasks is shown in Equation (3):

(3) Re=ρ·n·d2η

*ρ* is the density, *n* is the shaking frequency, and *η* is the dynamic viscosity.

It should be noted that, also at water‐like viscosity, unfavorable out‐of‐phase operating conditions can be encountered if shake flasks with excessively large baffles are employed. High viscosity and a high degree of baffling have the same negative effect [33].

## 3 Effects of Foam on the Oxygen Transfer Rate in Stirred Tank Fermenters and Shake Flasks

**FIGURE 1:**
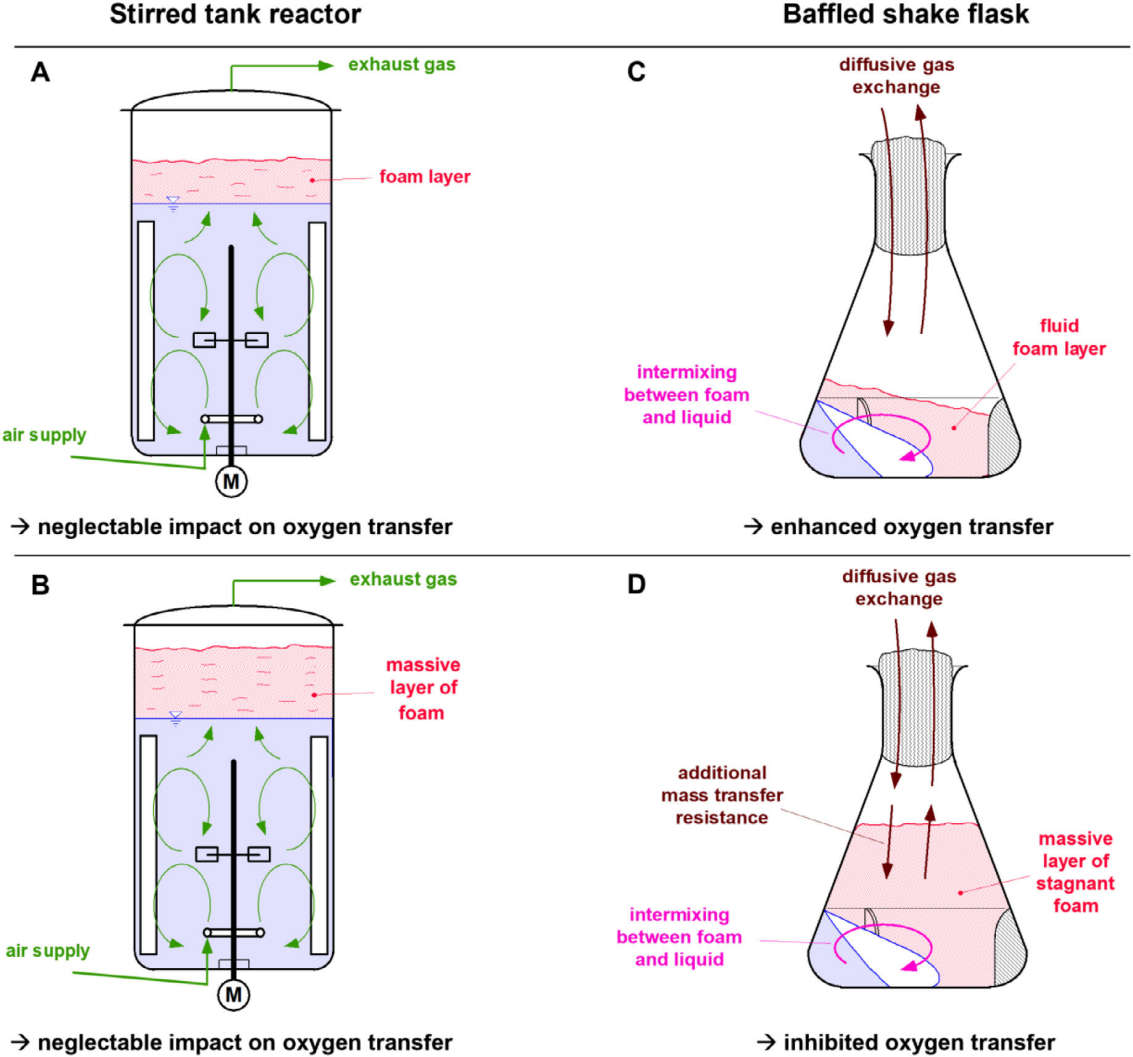
Impact of foam on the oxygen transfer rate in stirred tank reactors and in shake flasks. (A) Small quantities of foam hardly affect oxygen transfer in stirred tank reactors. (B) Even large quantities of foam do not affect oxygen transfer in stirred tank reactors, unless the foam spills over in the exhaust line. (C) In contrast, small quantities of fluid foam enhance oxygen transfer in shake flasks, as the foam provides additional mass transfer area. (D) Large quantities of stagnant foam reduce oxygen transfer in shake flasks, as an additional mass transfer resistance is created.

Foaming occurs both in stirred tank reactors and in shake flasks, but the effects of foam on the OTR are very different. The OTR, in general, can be described by Equation (4) [34]:

(4) $$\mathrm{OTR}=k_{L}\;a\cdot L_{O2}\cdot p_{\mathrm{abs}}\cdot \left(y_{O2}-y_{O2,L}\right)$$

where OTR is the oxygen transfer rate, *k*_L_ *a* is the volumetric mass transfer coefficient, *L*_O2_ is the oxygen solubility, *p*_abs_ is the absolute pressure, *y*_O2_ is the oxygen mole fraction in the liquid at the phase boundary, and *y*_O2,L_ is the oxygen mole fraction in the bulk liquid. The *k*_L_ *a* value can be expressed via Equation (5):

(5) $$k_{L}a=k_{L}\;\cdot a=k_{L}\;\cdot \frac{A}{V_{L}}$$

where *k*_L_ is the mass transfer coefficient, *a* is the volume‐specific mass transfer area, and *A* is the oxygen transfer area. Assuming *L*_O2_, *p*_abs_, *k*_L_, and *V*_L_ do not change during cultivation, the OTR is solely controlled by *A*, the oxygen transfer area. In the case of stirred tank fermenters, *A* mainly represents the entire surface area of all air bubbles in the fermenter. In the case of shake flasks, *A* represents the surface area of the rotating liquid. It is noteworthy that in shake flasks, the total liquid surface area *A* is composed of the surface area of the rotating bulk liquid and the thin liquid film on the glass wall. The effects of foam formation on the OTR in stirred tank reactors and in shake flasks are shown in Figure 1.

Figure 1A depicts the scenario of small quantities of foam in a stirred tank reactor. Foam in a stirred tank reactor accumulates on the surface of the liquid in the bioreactor headspace. A stirred tank reactor is an actively gassed system. Air is constantly introduced via a sparger into the fermentation broth; the air bubbles are finely dispersed by the stirrer and move upwards. According to Equations (4) and (5), the OTR is dependent on the oxygen transfer area *A*, which is mainly defined by the surface area of the finely dispersed air bubbles. A foam layer on top of the fermenter does not influence this mass transfer area (Figure 1A,B). Hence, the oxygen supply is not influenced, unless the foam clogs the exhaust line. This poses a risk of equipment damage and should, in any case, be avoided to ensure safe, sterile, and defined operation conditions. In contrast, a shake flask is a passively gassed cultivation device. The surface area of the rotating liquid represents the oxygen transfer area *A*. Small quantities of fluid foam and a constant intermixing between the rotating bulk liquid and the foam layer provide additional mass transfer area to the shake flask (Figure 1C). Consequently, the OTR_max_ in shake flasks increases. However, when the amount of foam increases and a massive layer of stagnant foam is formed, this effect reverses (see Figure 1D). A massive layer of stagnant foam adds an additional mass transfer resistance to the shake flask and, thus, decreases the OTR_max_. In contrast, in stirred tank fermenters, a massive layer of stagnant foam still has no direct influence on the oxygen transfer (Figure 1B), unless it spills over in the exhaust line.

## 4 Results and Discussion

**FIGURE 2:**
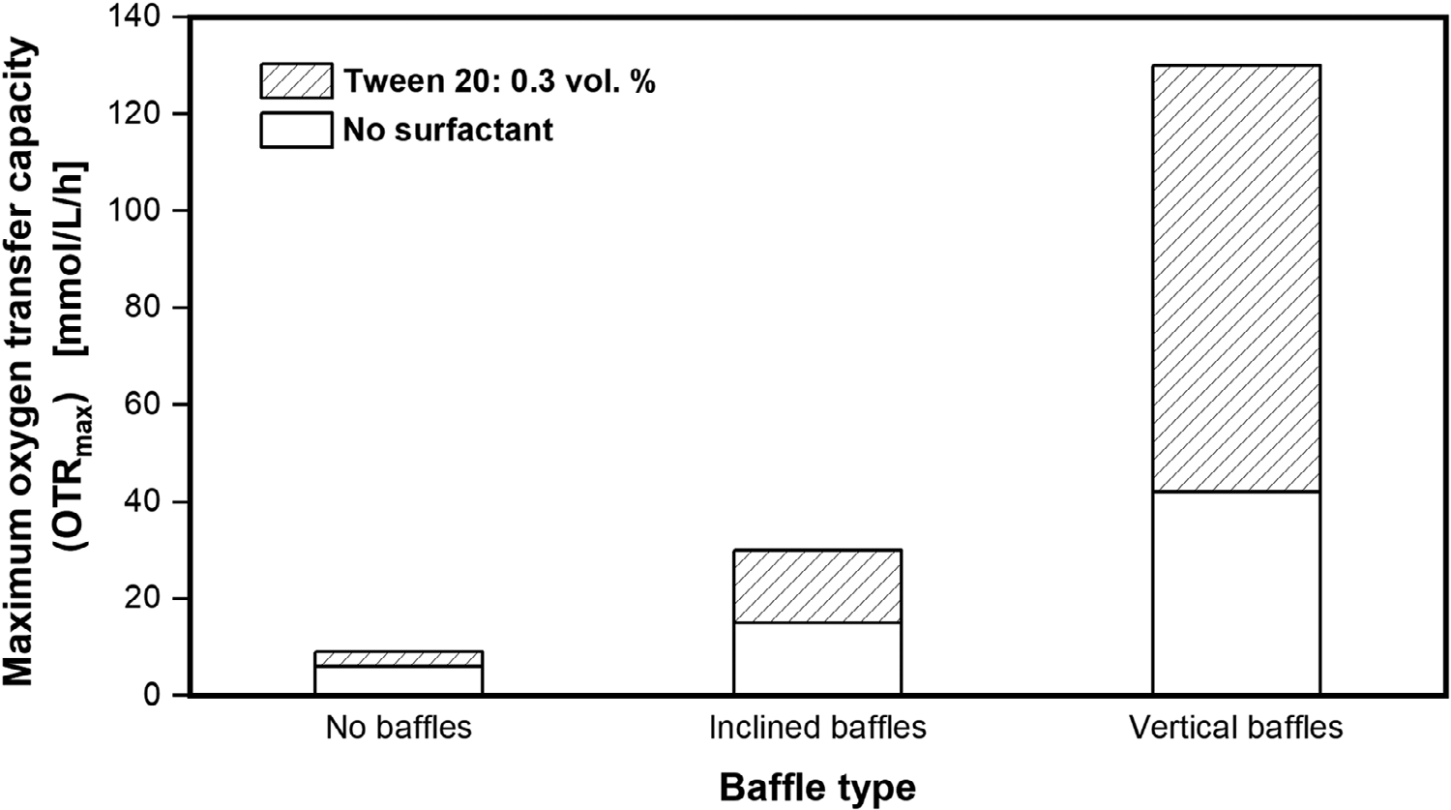
Comparison of different baffle types in shake flasks and their impact on the maximum oxygen transfer capacity (OTR_max_). Technical drawing of the baffle dimensions shown in Figure S1. Experimental conditions: 1 M sulfite system (0.1 M phosphate buffer, pH_Start_ = 8.0, 3.3 × 10^−7^ M CoSO_4_), *V*_F_ = 500 mL, *V*_L_ = 30 mL, *n* = 150 rpm, *d*_0_ = 50 mm, *T* = 22.5°C. Figure adapted from Maier [35].

To study the effect of foam formation on the OTR_max_ in shake flasks in more detail, the abiotic sulfite system was used. Tween 20 was added to the sulfite system to allow foam formation. The results for different types of baffled and non‐baffled shake flasks are shown in Figure 2.

**FIGURE 3:**
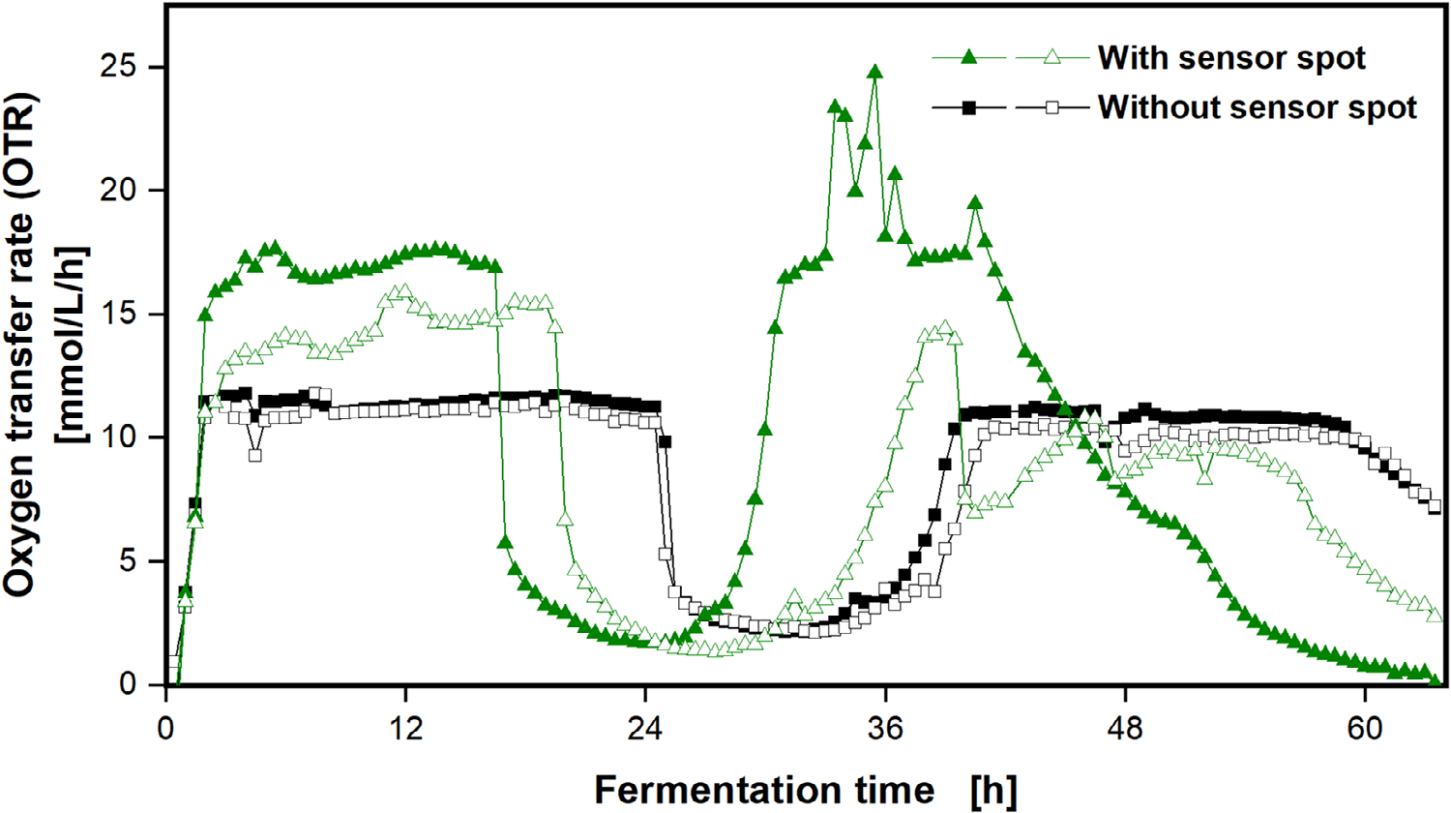
Online monitoring of oxygen transfer rate of an *Escherichia coli* VH33 cultivation with a RAMOS device. The sensor spots of the cultivation indicated by empty and filled green triangles were glued on the inner glass wall of the shake flask at the height of the largest inner diameter. Cultivation conditions: TB medium (5 g/L glycerol, 72 mM K_2_HPO_4_, 17 mM KH_2_PO_4_, pH_Start_ = 7), *V*_F_ = 500 mL, *V*_L_ = 100 mL, *n* = 250 rpm, *d*_0_ = 50 mm, *T* = 37°C.

In non‐baffled shake flasks, nearly no impact of the surfactant Tween 20 on the OTR is detected. This is expected, since non‐baffled shake flasks are known to prevent foaming [16]. In shake flasks with inclined or vertical baffles (see Figure S1), an overall increased OTR is observed. Baffles lead to a higher power input and, thus, increase the OTR_max_ [35]. When no surfactant is added to the sulfite system, and, thus, no foaming occurs, inclined baffles lead to a 2.5 times higher OTR_max_ (15 mmol/L/h), compared to non‐baffled shake flasks (6 mmol/L/h). Vertical baffles show a 7‐fold higher OTR_max_ (42 mmol/L/h), compared to non‐baffled shake flasks. When the surfactant Tween 20 is added to the sulfite system, foam formation in the baffled shake flasks occurs due to the turbulent liquid movement, which further increases the OTR_max_, as illustrated in Figure 1C. The OTR_max_ increases twofold (30 mmol/L/h) and threefold (130 mmol/L/h), for inclined baffles and vertical baffles, respectively. Therefore, the introduction of baffles into shake flasks under otherwise identical conditions has two separate effects on the gas/liquid mass transfer. First, the power input increases [35], unless with excessively large baffles, out‐of‐phase conditions are encountered [33]. A larger power input increases the gas/liquid mass transfer. Second, baffles generate foam, an effect that has been overlooked in the past and additionally increases the gas/liquid mass transfer. The results in Figure 2 demonstrate how foam in shake flasks can affect the OTR_max_ by provoking an additional mass transfer area. Non‐baffled shake flasks are commonly regarded to prevent foaming. However, under certain conditions, when sensor spots for online measurements are attached to the inside of the shake flask, a baffling effect and foaming may occur. In Figure 3, the results of an *E. coli* fermentation in 500 mL shake flasks are shown. In this experiment, two of the four shake flasks were equipped with sensor spots, glued to the inner glass wall of the shake flask at the height of the largest inner diameter (see Figure 3, open and filled green triangles). A picture of the sensor spots can be seen in Figures S2 and S3. These sensor spots are commonly used for online measurement of the pH value (data not shown) [36]. The OTR of the cultivations without sensor spots (Figure 3, open and filled black squares) exponentially increases and remains under oxygen limitation at an OTR of 11.5 mmol/L/h for approximately 24 h. Afterwards, the OTR drops and starts increasing again after 32 h and remains under another period of oxygen limitation for approximately 18 h. At the end of the cultivation, the OTR starts to decline. Both duplicates without sensor spots show a similar progression with low deviation from each other. In contrast, the cultivations equipped with a sensor spot show a significantly higher OTR. At the end of the exponential growth phase, during the period of oxygen limitation, the OTRs reach values of 17.5 mmol/L/h (green‐filled triangles) and 16 mmol/L/h (green empty triangles). The OTR_max_ during oxygen limitation of the foaming cultivation broth is about 4–6 mmol/L/h higher than the OTR_max_ of the non‐foaming cultivation. This phenomenon can be explained by the formation of foam in the non‐baffled 500 mL shake flasks. Pictures of the foaming cultivation broths can be seen in Figures S2 and S3. The tiny sensor spots, which are glued to the inside of the shake flask, act as small baffles and cause turbulent mixing in the cultivation broth. Since TB medium with additional Tween 20 as a surfactant was used, this medium has a strong tendency to foam. The fluid foam layer adds additional oxygen transfer area to the liquid in the shake flask and increases the OTR_max_. To the best of our knowledge, this is the first time, an increase in the OTR_max_ in a shake flask cultivation, due to foam formation, has been presented. The first phase of oxygen limitation of the foaming cultivation broths ends at different fermentation times between 17 and 20 h. This is 5–8 h earlier, compared to the non‐foaming cultivation broths, owing to the higher OTR_max_, resulting in a faster consumption of the first carbon source. At the end of the first oxygen limitation, both OTRs show an intermediate drop and start to rise again after approximately 24 h. Once more, the foaming cultivations with the sensor spots show significantly higher OTRs, compared to the non‐foaming cultivations. In the end, both OTRs decline. Another effect, next to the increase of the OTR_max_, related to the foam formation is an impact on the reproducibility. Foaming in shake flasks due to baffles is an undefined process. Hence, the amount of created foam of the duplicate cultivations with sensor spots differs (see Figure S2B). This leads to different OTR_max_ (see Figure 3, green‐filled triangles and green empty triangles). The higher OTR_max_ of the first foaming cultivation (green‐filled triangles) leads to faster consumption of the carbon source, compared to the second foaming cultivation (green empty triangles). This is further demonstrated by the culture with the green‐filled triangles ending earlier (after 16.5 h), as the OTR approaches zero, compared to the culture with the green open triangles (after 19.5 h). Thus, foaming has a severe impact on the reproducibility of oxygen‐limited fermentations in shake flasks and should be considered as a warning sign. In this case, foam formation was indicated by the OTR signal. Without online monitoring, the beginning of foaming would have remained unclear, in the first place, and would probably only have been noticed during sampling or at the end of the cultivation. Although an elevated OTR_max_ due to foaming might seem beneficial at first glance, it comes with a significant drawback of poor reproducibility.

**FIGURE 4:**
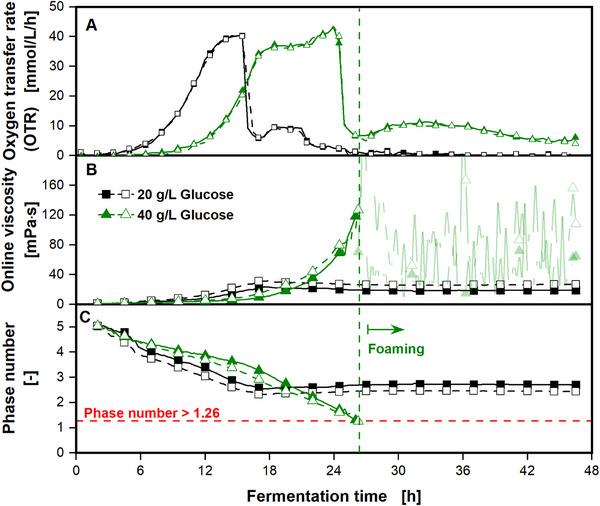
Online monitoring of oxygen transfer rate and online viscosity of a *Bacillus subtilis* P_xyl_ Δspo cultivation. (A) Online monitoring of oxygen transfer rate with a RAMOS device. (B) Online monitoring of viscosity, compared to the offline viscosity, is measured from offline samples with a rheometer. (C) Phase number calculated from online viscosity. The horizontal red dashed line marks the critical phase number of 1.26. The vertical green dashed line indicates the beginning of foaming of the cultivation with an initial glucose concentration of 40 g/L (green triangles). For clarity, only every third (A) and tenth data point (B) and (C) is shown. Cultivation conditions: Modified V3 glucose minimal medium (20–40 g/L glucose, 0.2–0.4 M MOPS buffer, pH_Start_ = 8.1), *V*_F_ = 250 mL, *V*_L_ = 20 mL, *n* = 350 rpm, *d*_0_ = 50 mm, *T* = 37°C. Offline viscosity was measured in single measurement. Figure adapted from Hoffmann [38].

In the following, it is presented how elevated viscosities during cultivations in non‐baffled shake flasks can induce foam formation. Figure 4 shows a cultivation of *B. subtilis*. This organism produces γ‐PGA, a biopolymer leading to elevated viscosities. The microorganism was cultivated under two different conditions. Figure 4A shows the OTRs of the cultivations with 20 g/L of initial glucose (black open and filled squares) and with 40 g/L of initial glucose (green open and filled triangles). The reproducibility of both duplicates is excellent. Both curves show an increase in the OTR until a plateau at about 37 mmol/L/h is reached. The cultivations with an initial glucose concentration of 20 g/L reach the plateau earlier, compared to the cultivations with an initial glucose concentration of 40 g/L, probably due to the lower osmolality, leading to a shorter lag phase. Afterward, a sudden decrease in the OTR is visible with a subsequent slight increase, followed by a constant decrease until the end of the fermentation. In Figure 4B, the online measured viscosity is depicted. The online viscosity of the cultivations with an initial glucose concentration of only 20 g/L increases up to approximately 30 mPa·s until a fermentation time of 18 h and remains at this level. The online viscosity of the cultivations with an initial glucose concentration of 40 g/L is nearly exponentially increasing, until reaching a viscosity of 120 mPa·s after a cultivation time of 26 h. Afterward, foaming of both duplicates with an initial glucose concentration of 40 g/L occurs, indicated by the vertical green dashed line. Due to foaming, online measurements of the viscosity are no longer reliable. The foam interferes with the transmitted light measurements and prevents a reliable detection of the leading edge of the rotating bulk liquid. A picture of the foaming cultivation broth is shown in Figure S4. No foam formation is visible in both cultivations with an initial glucose concentration of 20 g/L. In Figure 4C, the phase number, calculated from the online viscosity measurements, is presented. The phase numbers are initially decreasing due to increasing viscosities. The phase numbers of the cultivations with an initial glucose concentration of 20 g/L remain at a phase number of about 2.5, and, thus, at in‐phase conditions throughout the entire cultivation. In contrast, the phase numbers of the cultivations with an initial glucose concentration of 40 g/L are gradually decreasing. After a cultivation time of 26 h, the phase numbers pass the critical value of 1.26, indicating out‐of‐phase conditions. This is the exact time point when foaming occurs. This suggests a relation between foaming and the out‐of‐phase phenomenon. In the previous experiments, foaming was either caused by a baffle (Figure 2) or by a sensor spot, also with a baffling effect (Figure 3). In both cases, mechanical obstacles interfered with the laminar fluid flow and caused turbulence, resulting in foaming. However, in this case of the cultivation of *B. subtilis*, a non‐baffled shake flask without modifications was used. Hence, the liquid itself must have acted as a mechanical obstacle or baffle. Due to the increasing viscosity, the phase number of the system is slowly decreasing, leading to out‐of‐phase conditions. During this gradual transition, parts of the rotating bulk liquid transient from in‐phase conditions to out‐of‐phase conditions. These parts of the viscous liquid presumably stick to the flask bottom and act as an obstacle and, therefore, as a baffle for the remaining rotating bulk liquid [37]. This might introduce turbulence and air bubbles in the viscous cultivation broth and, hence, promote foam formation. Contrary to the foaming of the cultivation of *E. coli* (Figure 3), the beginning of foam formation is not evident from the OTR signal, since the cultivation is not oxygen‐limited. Instead, the beginning of foaming is made apparent by the online monitoring of the viscosity. Again, without online monitoring, the exact time point of foaming would have most likely remained unclear.

**FIGURE 5:**
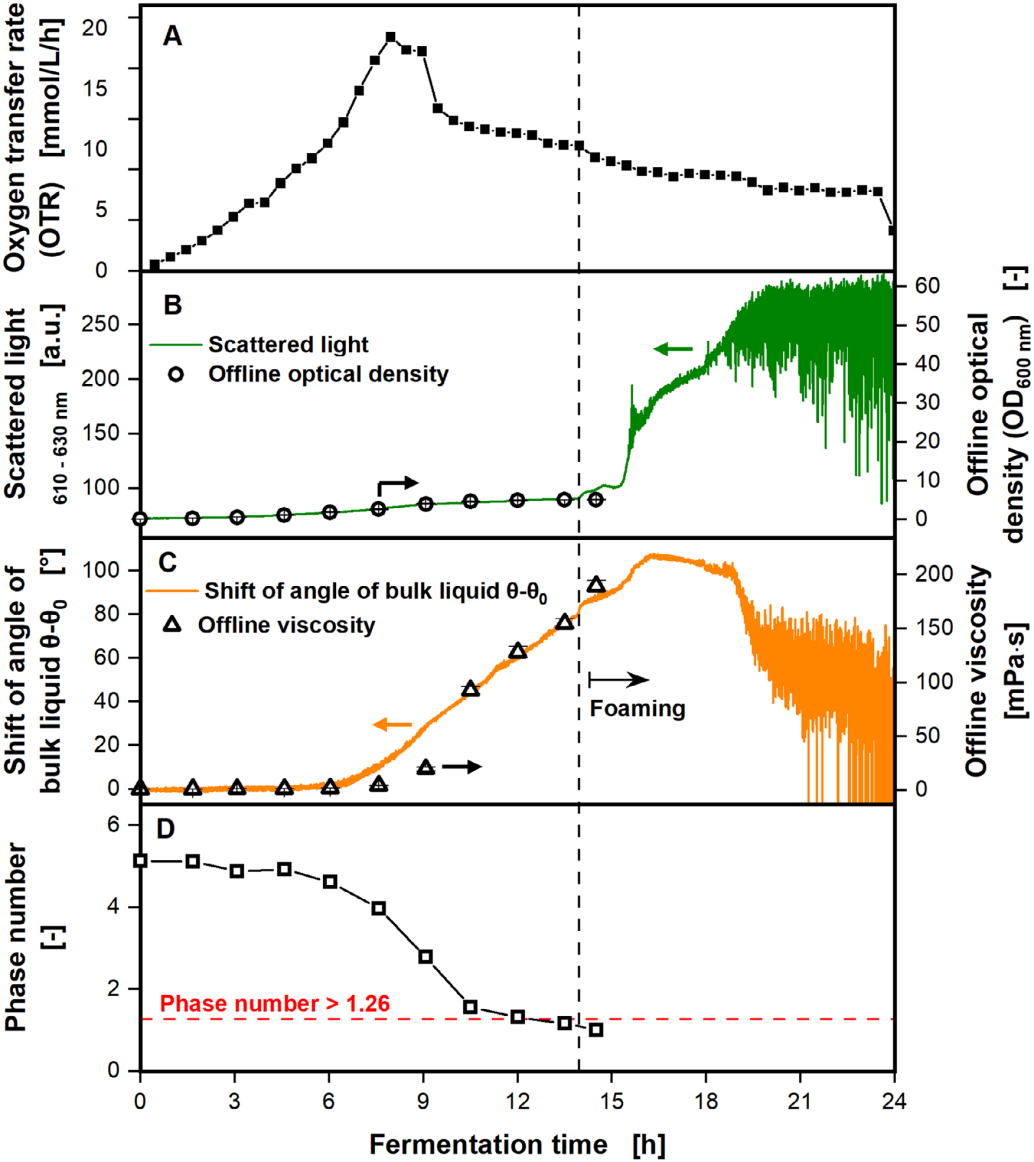
Online monitoring of oxygen transfer rate, scattered light, and shift of angle of the bulk liquid relative to the direction of the centrifugal acceleration *θ–θ*_0_ of a *Paenibacillus polymyxa* DSM365 cultivation. (A) Online monitoring of oxygen transfer rate with a RAMOS device. (B) Online monitoring of scattered light, compared to optical density measurements from offline samples at 600 nm. (C) Online monitoring of the shift of angle of the bulk liquid *θ–θ*_0_, compared to the offline viscosity, measured from offline samples with a rheometer. (D) Phase number calculated from offline measured viscosity (C). The horizontal red dashed line marks the critical phase number of 1.26. The vertical black dashed line marks the beginning of foaming, indicated by the sudden increase of the scattered light signal. Cultivation conditions: MM1P100 medium (30 g/L glucose, 12 mM KH_2_PO_4_, pH_Start_ = 7); 0.2 mg/mL fluorescent Oxnano nanoparticles; *V*_F_ = 250 mL, *V*_L_ = 30 mL, *n* = 200 rpm, *d*_0_ = 50 mm, *T* = 30°C. Standard deviation is barely visible, as it is smaller than the data points. Figure adapted from Dinter et al. [19].

The phenomenon of foam formation in non‐baffled shake flasks due to increasing viscosities, leading to out‐of‐phase conditions, was first described by Dinter et al. [19]. They cultivated *P. polymyxa* in shake flasks with online monitoring of dissolved oxygen tension, pH value, scattered light, and viscosity. *P. polymyxa* produces extracellular polysaccharides, thus increasing the viscosity of the cultivation broth [38]. Figure 5 shows an adapted figure from the cultivation shown by Dinter et al. [19].

The OTR of *P. polymyxa* is depicted in Figure 5A. The OTR is increasing until a value of 20 mmol/L/h after a cultivation time of 8 h. Subsequently, the OTR is decreasing until the end of the fermentation. In Figure 5B, the online scattered light is compared to the offline optical density. Until a cultivation time of 14 h, the online measured scattered light signal correlates well with the offline measured optical density. Afterward, the cultivation broth starts foaming, indicated by the vertical black dashed line. Pictures of the foaming cultivation broths are shown in Figure S5. Due to the beginning of foaming, the scattered light signal increases and does not correlate with the offline optical density anymore. After a fermentation time of 15 h, the scattered light signal increases rapidly and strongly fluctuates due to excessive foam formation. In Figure 5C, the shift of angle of bulk liquid *θ–θ*_0_ is compared to the offline viscosity. Both measures correlate well with each other until foaming occurs. Afterwards, the measured shift of angle of the bulk liquid *θ–θ*_0_ deviates from the offline viscosity, due to the foaming. In Figure 5D, the phase number is depicted. The phase number decreases throughout the cultivation and finally falls below the critical threshold of 1.26. As in the cases discussed before, foaming occurs when the phase number indicates out‐of‐phase conditions. To prove the relation between foam formation in non‐baffled shake flasks and the out‐of‐phase phenomenon, three additional cultivations of *P. polymyxa* were performed (see Figures S6–S8). In all three cultivations, the exact moment of foam formation was indicated by a sudden increase in the online monitored scattered light signals. Consistently, foaming always occurred when the phase number indicated out‐of‐phase conditions. This clearly demonstrates the connection between the out‐of‐phase phenomenon and foam formation in non‐baffled shake flasks. Online monitoring of the scattered light proved a reliable measure to indicate the time point of foaming.

## 5 Conclusions

In this work, the causes of foam formation in baffled and non‐baffled shake flasks and their impact on the oxygen transfer were demonstrated. It has been shown that the introduction of baffles into shake flasks has two separate positive effects on the gas/liquid mass transfer. Baffles increase the power input (unless out‐of‐phase flow conditions are encountered), which enhances the turbulent flow intensity and, therefore, mass transfer. In addition, baffles generate foam, which enlarges the mass transfer area. Until now, it was assumed that in non‐baffled shake flasks, no foaming problems are to be expected. However, in this study, it could be shown that foaming in non‐baffled shake flasks is possible and can confuse experimental results. Thus, foam formation in non‐baffled shake flasks should be considered as a warning signal. It could be shown that non‐baffled shake flasks with internals, such as tiny sensor spots, can cause foam formation. Foaming in shake flasks can increase the OTR and, thus, severely affect the cultivation conditions in oxygen‐limited cultivations. Due to the elevated OTR_max_ in a foaming system, comparisons to non‐foaming shake flask cultivations can be problematic, as the cultivation conditions over time may severely differ. Additionally, undefined foaming in shake flasks can lead to a strongly reduced reproducibility. Moreover, a direct relation between the out‐of‐phase phenomenon and foam formation in non‐baffled shake flasks was demonstrated. This is of particular importance when cultivating microorganisms at elevated viscosities. Online monitoring systems, like scattered light measurements, are very useful to indicate the exact time point of foaming. When working at elevated viscosities, in‐phase cultivation conditions should be ensured to avoid foam formation.

## Nomenclature

*a*
: volume‐specific gas/liquid mass transfer area [m^2^]
*A*
: oxygen transfer area [m^2^/m^3^]
*d*
: largest inner flask diameter [m]
*d*_0_
: shaking diameter [m]
*k*_L_
: mass transfer coefficient [m/h]
*k*_L_ *a*
: volumetric mass transfer coefficient [h^−1^]
LB: lysogeny broth
*L*_O2_
: oxygen solubility [mol/L/bar]
*n*
: shaking frequency [s^−1^]
OD_600_
: optical density at a wavelength of 600 nm [‐]
OTR: oxygen transfer rate [mol/L/h]
OTR_max_
: maximum oxygen transfer capacity [mol/L/h]
*P*_abs_
: absolute pressure [bar]
Ph: phase number [‐]
RAMOS: Respiration Activity Monitoring System
Re: Reynolds number [‐]
Re_film_
: Reynolds film number [‐]
*T*
: temperature [°C]
TB: terrific broth
*V*_L_
: filling volume [m^3^]
ViMOS: Viscosity Monitoring Online System
*y*_O2_
: oxygen mole fraction in the liquid at the phase boundary [mol/mol]
*y*_O2,L_
: oxygen mole fraction in the bulk liquid [mol/mol]
γ‐PGA: poly‐γ‐glutamic‐acid
*η*
: dynamic viscosity [Pa∙s]
*Θ*
: angle of bulk liquid relative to the direction of the centrifugal acceleration [‐]
*Θ*_0_
: angle of bulk liquid at the beginning of the cultivation [‐]
*ρ*
: density [kg/m^3^]

## Author Contributions

**David Vonester:** conceptualization, methodology, investigation, formal analysis, writing – original draft, writing – review and editing, visualization. **Thomas Palmen:** investigation, formal analysis. **Kyra Hoffmann:** investigation, formal analysis. **Lena Regestein:** investigation, formal analysis. **Ulrike Richter:** investigation, formal analysis. **Anna‐Lena Altenhoff:** investigation, formal analysis. **Maximilian Hoffmann:** investigation, **Yulia Radeva:** investigation, formal analysis. **Jochen Büchs:** conceptualization, methodology, formal analysis, supervision, writing – review and editing, project administration, funding acquisition. **Jørgen Barsett Magnus:** formal analysis, supervision, resources, writing – review and editing, project administration. All authors have read and approved the final version of the manuscript.

## Conflicts of Interest

The authors declare no conflicts of interest.

## Supporting information




**Supporting File 1**: elsc70057‐sup‐0001‐SuppMat.pdf

## Data Availability

Data will be made available on request.

## References

[1] F. Vardar‐Sukan , “Foaming: Consequences, Prevention and Destruction,” Biotechnology Advances 16 (1998): 913–948, 10.1016/S0734-9750(98)00010-X.

[2] P. Walstra , “Principles of Foam Formation and Stability,” in Foams: Physics, Chemistry and Structure, ed. A. Wilson (Springer, 1989), 1–15, 10.1007/978-1-4471-3807-5_1.

[3] W. Drenckhan and S. Hutzler , “Structure and Energy of Liquid Foams,” Advances in Colloid and Interface Science 224 (2015): 1–16, 10.1016/j.cis.2015.05.004. 26233494

[4] I. Cantat , S. Cohen‐Addad , F. Elias , et al., Foams: Structure and Dynamics (Oxford University Press, 2013), 10.1093/acprof:oso/9780199662890.001.0001.

[5] K. Małysa , “Wet Foams: Formation, Properties and Mechanism of Stability,” Advances in Colloid and Interface Science 40 (1992): 37–83, 10.1016/0001-8686(92)80071-5.

[6] F. Vardar‐Sukan , “Foaming and Its Control in Bioprocesses,” in Recent Advances in Biotechnology, ed. F. Vardar‐Sukan , Ş. S. Sukan (Springer, 1992), 113–146, 10.1007/978-94-011-2468-3_6.

[7] G. St‐Pierre Lemieux , D. Groleau , and P. Proulx , “Introduction on Foam and Its Impact in Bioreactors,” Canadian Journal of Biotechnology 3 (2019): 143–157, 10.24870/cjb.2019-000131.

[8] T. Tiso , P. Demling , T. Karmainski , et al., “Foam Control in Biotechnological Processes—Challenges and Opportunities,” Discover Chemical Engineering 4 (2024): 2, 10.1007/s43938-023-00039-0.

[9] D. Weuster‐Botz , J. Altenbach‐Rehm , and A. Hawrylenko , “Process‐Engineering Characterization of Small‐Scale Bubble Columns for Microbial Process Development,” Bioprocess and Biosystems Engineering 24 (2001): 3–11, 10.1007/s004490100222.

[10] C. C. Blesken , T. Strümpfler , T. Tiso , and L. M. Blank , “Uncoupling Foam Fractionation and Foam Adsorption for Enhanced Biosurfactant Synthesis and Recovery,” Microorganisms 8 (2020): 2029, 10.3390/microorganisms8122029. 33353027 PMC7766737

[11] V. Rangarajan , G. Dhanarajan , and R. Sen , “Bioprocess Design for Selective Enhancement of Fengycin Production by a Marine Isolate *Bacillus Megaterium* ,” Biochemical Engineering Journal 99 (2015): 147–155, 10.1016/j.bej.2015.03.016.

[12] S. D. Ganjave , H. Dodia , A. V. Sunder , S. Madhu , and P. P. Wangikar , “High Cell Density Cultivation of *E. coli* in Shake Flasks for the Production of Recombinant Proteins,” Biotechnology Reports 33 (2022): e00694, 10.1016/j.btre.2021.e00694. 35004235 PMC8718739

[13] J. A. Running and K. Bansal , “Oxygen Transfer Rates in Shaken Culture Vessels From Fernbach Flasks to Microtiter Plates,” Biotechnology and Bioengineering 113 (2016): 1729–1735, 10.1002/bit.25938. 26806816

[14] H. Toyosaki , T. Naritomi , A. Seto , M. Matsuoka , T. Tsuchida , and F. Yoshinaga , “Screening of Bacterial Cellulose‐Producing Acetobacter Strains Suitable for Agitated Culture,” Bioscience, Biotechnology, and Biochemistry 59 (1995): 1498–1502, 10.1271/bbb.59.1498.

[15] K. Ukkonen , A. Vasala , H. Ojamo , and P. Neubauer , “High‐Yield Production of Biologically Active Recombinant Protein in Shake Flask Culture by Combination of Enzyme‐Based Glucose Delivery and Increased Oxygen Transfer,” Microbial Cell Factories 10 (2011): 107, 10.1186/1475-2859-10-107. 22152005 PMC3258199

[16] J. Büchs , “Introduction to Advantages and Problems of Shaken Cultures,” Biochemical Engineering Journal 7 (2001): 91–98, 10.1016/S1369-703X(00)00106-6. 11173295

[17] H. Razafindralambo , M. Paquot , A. Baniel , et al., “Foaming Properties of Surfactin, a Lipopeptide Biosurfactant From *Bacillus Subtilis* ,” Journal of the American Oil Chemists Society 73 (1996): 149–151, 10.1007/BF02523463.

[18] J. S. Guez , C. H. Müller , P. M. Danze , J. Büchs , and P. Jacques , “Respiration Activity Monitoring System (RAMOS), an Efficient Tool to Study the Influence of the Oxygen Transfer Rate on the Synthesis of Lipopeptide by *Bacillus Subtilis* ATCC6633,” Journal of Biotechnology 134 (2008): 121–126, 10.1016/j.jbiotec.2008.01.003. 18282625

[19] C. Dinter , D. Vonester , D. Flitsch , et al., “Combined Optical Measurement of Dissolved Oxygen Tension (DOT), pH Value, Biomass and Viscosity in Shake Flasks,” Biochemical Engineering Journal 212 (2024): 109515, 10.1016/j.bej.2024.109515.

[20] S. Tesche , R. Rösemeier‐Scheumann , J. Lohr , R. Hanke , J. Büchs , and R. Krull , “Salt‐Enhanced Cultivation as a Morphology Engineering Tool for Filamentous Actinomycetes: Increased Production of Labyrinthopeptin A1 in *Actinomadura Namibiensis* ,” Engineering in Life Sciences 19 (2019): 781–794, 10.1002/elsc.201900036. 32624971 PMC6999293

[21] K. Meier , W. Klöckner , B. Bonhage , E. Antonov , L. Regestein , and J. Büchs , “Correlation for the Maximum Oxygen Transfer Capacity in Shake Flasks for a Wide Range of Operating Conditions and for Different Culture Media,” Biochemical Engineering Journal 109 (2016): 228–235, 10.1016/j.bej.2016.01.014.

[22] M. Losen , B. Frölich , M. Pohl , and J. Büchs , “Effect of Oxygen Limitation and Medium Composition on *Escherichia coli* Fermentation in Shake‐Flask Cultures,” Biotechnology Progress 20 (2004): 1062–1068, 10.1021/bp034282t. 15296430

[23] K. Hoffmann , B. Halmschlag , S. Briel , et al., “Online Measurement of the Viscosity in Shake Flasks Enables Monitoring of γ‐PGA Production in Depolymerase Knockout Mutants of *Bacillus Subtilis* With the Phosphate‐Starvation Inducible Promoter Ppst,” Biotechnology Progress 39 (2023):e3293, 10.1002/btpr.3293. 36081345

[24] B. Halmschlag , K. Hoffmann , R. Hanke , et al., “Comparison of Isomerase and Weimberg Pathway for γ‐PGA Production From Xylose by Engineered *Bacillus subtilis* ,” Frontiers in Bioengineering and Biotechnology 7 (2020): 476, 10.3389/fbioe.2019.00476. 32039180 PMC6985040

[25] M. Sieben , Characterization of the Permeability of Sealing Tapes and Development of a Viscosity Measuring Technique in Shaken Reactors (Rheinisch‐Westfälische Technische Hochschule Aachen, 2017).

[26] T. Anderlei and J. Büchs , “Device for Sterile Online Measurement of the Oxygen Transfer Rate in Shaking Flasks,” Biochemical Engineering Journal 7 (2001): 157–162, 10.1016/S1369-703X(00)00116-9. 11173305

[27] T. Anderlei , W. Zang , M. Papaspyrou , and J. Büchs , “Online Respiration Activity Measurement (OTR, CTR, RQ) in Shake Flasks,” Biochemical Engineering Journal 17 (2004): 187–194, 10.1016/S1369-703X(03)00181-5.

[28] M. Sieben , R. Hanke , and J. Büchs , “Contact‐Free Determination of Viscosity in Multiple Parallel Samples,” Scientific Reports 9 (2019): 8335, 10.1038/s41598-019-44859-z. 31171822 PMC6554296

[29] H. Giese , W. Klöckner , C. Peña , et al., “Effective Shear Rates in Shake Flasks,” Chemical Engineering Science 118 (2014): 102–113, 10.1016/j.ces.2014.07.037.

[30] J. Büchs , U. Maier , C. Milbradt , and B. Zoels , “Power Consumption in Shaking Flasks on Rotary Shaking Machines: I. Power Consumption Measurement in Unbaffled Flasks at Low Liquid Viscosity,” Biotechnology and Bioengineering 68 (2000): 589–593, 10.1002/(SICI)1097-0290(20000620)68:6<589::AID-BIT1>3.0.CO;2-J. 10799983

[31] J. Büchs , U. Maier , C. Milbradt , and B. Zoels , “Power Consumption in Shaking Flasks on Rotary Shaking Machines: II. Nondimensional Description of Specific Power Consumption and Flow Regimes in Unbaffled Flasks at Elevated Liquid Viscosity,” Biotechnology and Bioengineering 68 (2000): 594–601, 10.1002/(SICI)1097-0290(20000620)68:6<594::AID-BIT2>3.0.CO;2-U. 10799984

[32] A. Azizan , M. Sieben , G. Wandrey , and J. Büchs , “Reassessing the Out‐of‐Phase Phenomenon in Shake Flasks by Evaluating the Angle‐Dependent Liquid Distribution Relative to the Direction of the Centrifugal Acceleration,” Biotechnology and Bioengineering 116 (2019): 2983–2995, 10.1002/bit.27132. 31350917

[33] J. Büchs , S. Lotter , and C. Milbradt , “Out‐of‐Phase Operating Conditions, a Hitherto Unknown Phenomenon in Shaking Bioreactors,” Biochemical Engineering Journal 7 (2001): 135–141, 10.1016/S1369-703X(00)00113-3. 11173302

[34] U. Maier , M. Losen , and J. Büchs , “Advances in Understanding and Modeling the Gas‐Liquid Mass Transfer in Shake Flasks,” Biochemical Engineering Journal 17 (2004): 155–167, 10.1016/S1369-703X(03)00174-8.

[35] C. P. Peter , Y. Suzuki , K. Rachinskiy , S. Lotter , and J. Büchs , “Volumetric Power Consumption in Baffled Shake Flasks,”Chemical Engineering Science 61 (2006): 3771–3779, 10.1016/j.ces.2005.12.020.

[36] M. Scheidle , J. Klinger , and J. Büchs , “Combination of On‐Line pH and Oxygen Transfer Rate Measurement in Shake Flasks by Fiber Optical Technique and Respiration Activity MOnitoring System (RAMOS),” Sensors 7 (2007): 3472–3480, 10.3390/s7123472. 28903306 PMC3841907

[37] S. Lotter and J. Büchs , “Utilization of Specific Power Input Measurements for Optimization of Culture Conditions in Shaking Flasks,” Biochemical Engineering Journal 17 (2004): 195–203, 10.1016/S1369-703X(03)00178-5.

[38] C. Schilling , M. Gansbiller , B. Rühmann , V. Sieber , and J. Schmid , “Rheological Characterization of Artificial Paenan Compositions Produced by *Paenibacillus polymyxa* DSM 365,” Carbohydrate Polymers 320 (2023): 121243, 10.1016/j.carbpol.2023.121243. 37659800

