# Supplementary material for: Foam Formation in Shake Flasks and Its Consequences
Source: Eng Life Sci. 2025 Oct 22;25(10):e70057. doi: 10.1002/elsc.70057 (PMC12541551; doi:10.1002/elsc.70057)
Supplement: Supplementary file 1 — Supporting File 1: elsc70057‐sup‐0001‐SuppMat.pdf [file ELSC-25-e70057-s001.pdf]

Foam formation in shake flasks and its consequences – Supplementary Material

Supplementary Table S1 Composition of TB-medium for cultivation of *Escherichia coli.*

**Substance**	**Concentration**
Glycerol	5.0 g/L
Tryptone	12.0 g/L
Yeast extract	24.0 g/L
Dipotassium hydrogen phosphate	12.54 g/L
Potassium dihydrogen phosphate	2.31 g/L

The solution was autoclaved at 121 °C for 20 min.

Supplementary Table S2 Composition of lysogeny broth (LB) medium for cultivation of the first pre-culture of *Bacillus subtilis.*

**Substance**	**Concentration**
Peptone	10 g/L
Yeast extract	5 g/L
Sodium chloride	5 g/L

The solution was autoclaved at 121 °C for 20 min.

Supplementary Table S3 Composition of modified V3 glucose minimal medium for cultivation of the second pre-culture and main-culture of *Bacillus subtilis.*

**Substance**	**Concentration**
Glucose	20 – 40 g/L
Xylose	1 g/L (for main culture)
Magnesium sulfate heptahydrate	1.01 g/L
Manganese(II) chloride tetrahydrate	0.05 g/L
Calcium chloride tetrahydrate	0.026 g/L
Ammonium sulfate	15 g/L
Cobalt(II) chloride hexahydrate	0.53 mg/L
Zinc chloride	0.26 mg/L
Boric acid	0.01 mg/L
Nickel(II) sulfate hexahydrate	0.66 mg/L
Copper(II) sulfate pentahydrate	0.31 mg/L
Sodium molybdate dihydrate	0.65 mg/L
Iron(II) sulfate heptahydrate	0.05 g/L
MOPS, free acid	41.85 g/L – 83.7 g/L (200 mM – 400 mM)
Dipotassium hydrogen phosphate	3.4 g/L

Every substance was added from sterile stock solutions in the same sequence listed above, to avoid precipitation. The initial pH was set to 8.1 using 5 M NaOH.

Supplementary Table S4 Composition of the MM1P100 medium for cultivation of *Paenibacillus polymyxa.*

**Substance**	**Concentration**
Glucose	30 g/L
Casein peptone (Carl Roth GmbH & Co.)	5 g/L
Magnesiumsulfate heptahydrate	1.33 g/L
Potassium dihydrogen phosphate	1.67 g/L
Calciumchloride dihydrate	0.05 g/L
Vitamin solution RPMI 1640 (100x) (Sigma-Aldrich)	50 mL/L
Trace elements solution (1000x)	1 mL/L

All solutions were prepared as individual stock solutions. The pH value of the potassium dihydrogen phosphate solution was adjusted to 7.0 by using potassium hydroxide. The trace elements solution and potassium dihydrogen phosphate solution were sterile filtrated. The vitamin solution was purchased sterile. All other solutions were autoclaved at 121 °C for 20 min.

Supplementary Table S5 Composition of the trace elements solution for MM1P100 medium for cultivation of *Paenibacillus polymyxa.*

**Substance**	**Concentration**
Iron (II) sulfate heptahydrate	2.5 g/L
Sodium tartrate dihydrate	2.1 g/L
Manganese (II) chloride tetrahydrate	1.8 g/L
Cobalt (II) chloride hexahydrate	0.075 g/L
Copper (II) sulfate heptahydrate	0.031 g/L
Boric acid	0.258 g/L
Sodium molybdate	0.023 g/L
Zinc chloride	0.021 g/L

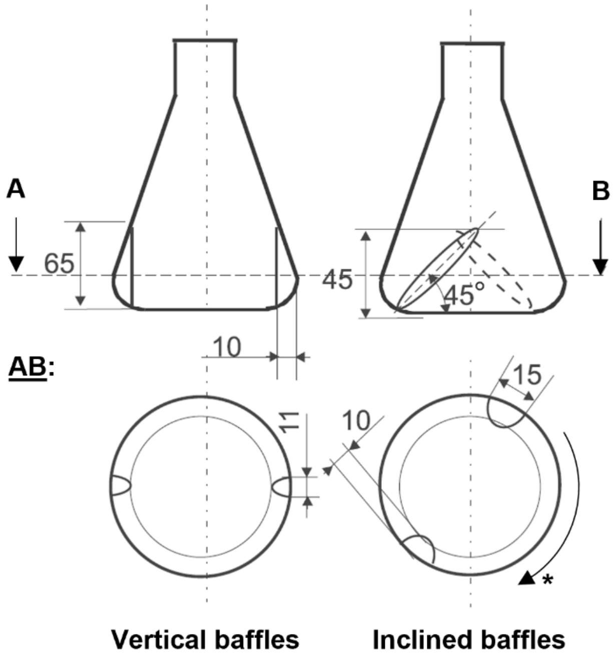

Supplementary Fig. S1 Technical drawing of the baffle dimensions of the shake flasks shown in Fig. 2. Black arrow with asterisk indicates direction of the rotating liquid movement. Figure adapted from Maier [35].

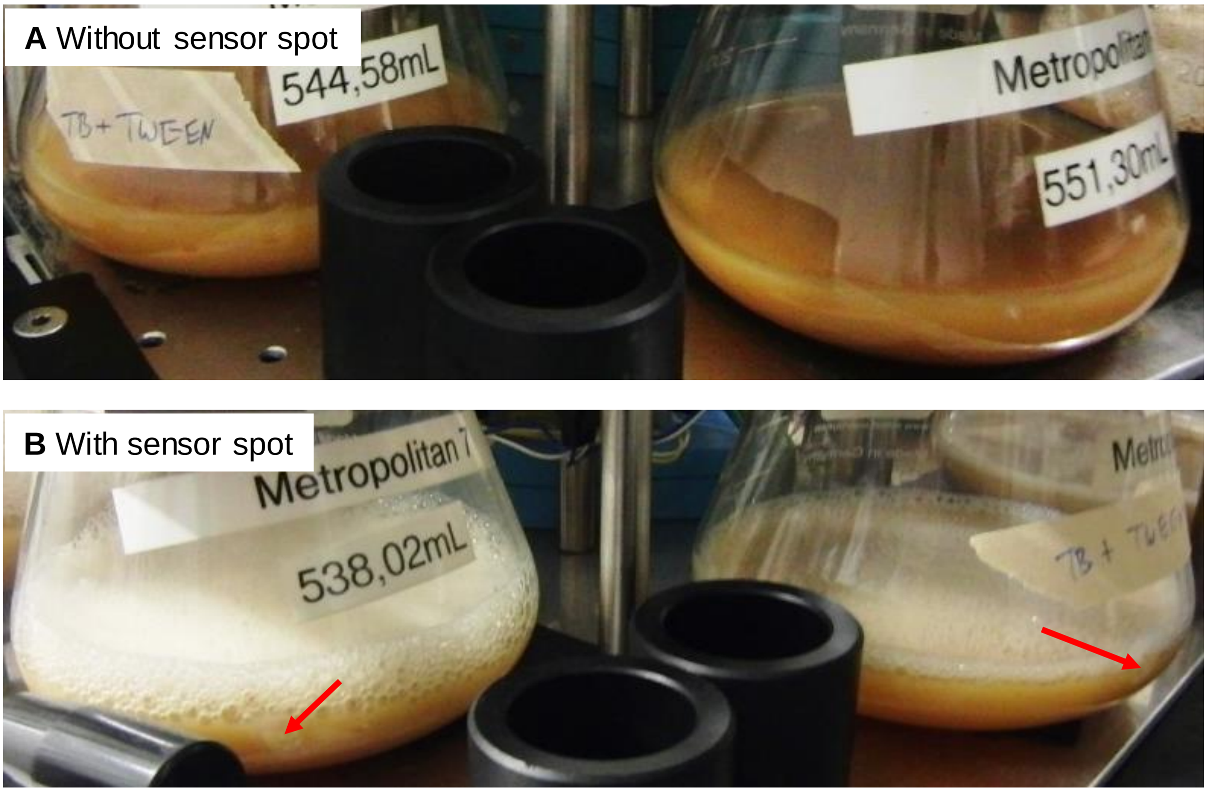

Supplementary Fig. S2 Pictures of the cultivation broth of *Escherichia coli* VH33 from Fig. 3. (A) Cultivation without sensor spots. (B) Cultivation with sensor spots. The sensor spots were glued to the inner glass wall of the shake flask at the height of the largest inner diameter. Pictures were taken after a cultivation time of 15.5 h. Red arrows indicate the position of the sensor spots. A close-up view of the sensor spot is shown in Supplementary Fig. S3. Cultivation conditions: TB medium (5 g/L glycerol, 72 mM K_2HPO_4, 17 mM KH_2PO_4, pH_{Start} = 7.5), flask volume (V_F) = 500 mL, filling volume (V_L) = 100 mL, shaking frequency (n) = 250 rpm, shaking diameter (d_0) = 50 mm, temperature (T) = 37 °C.

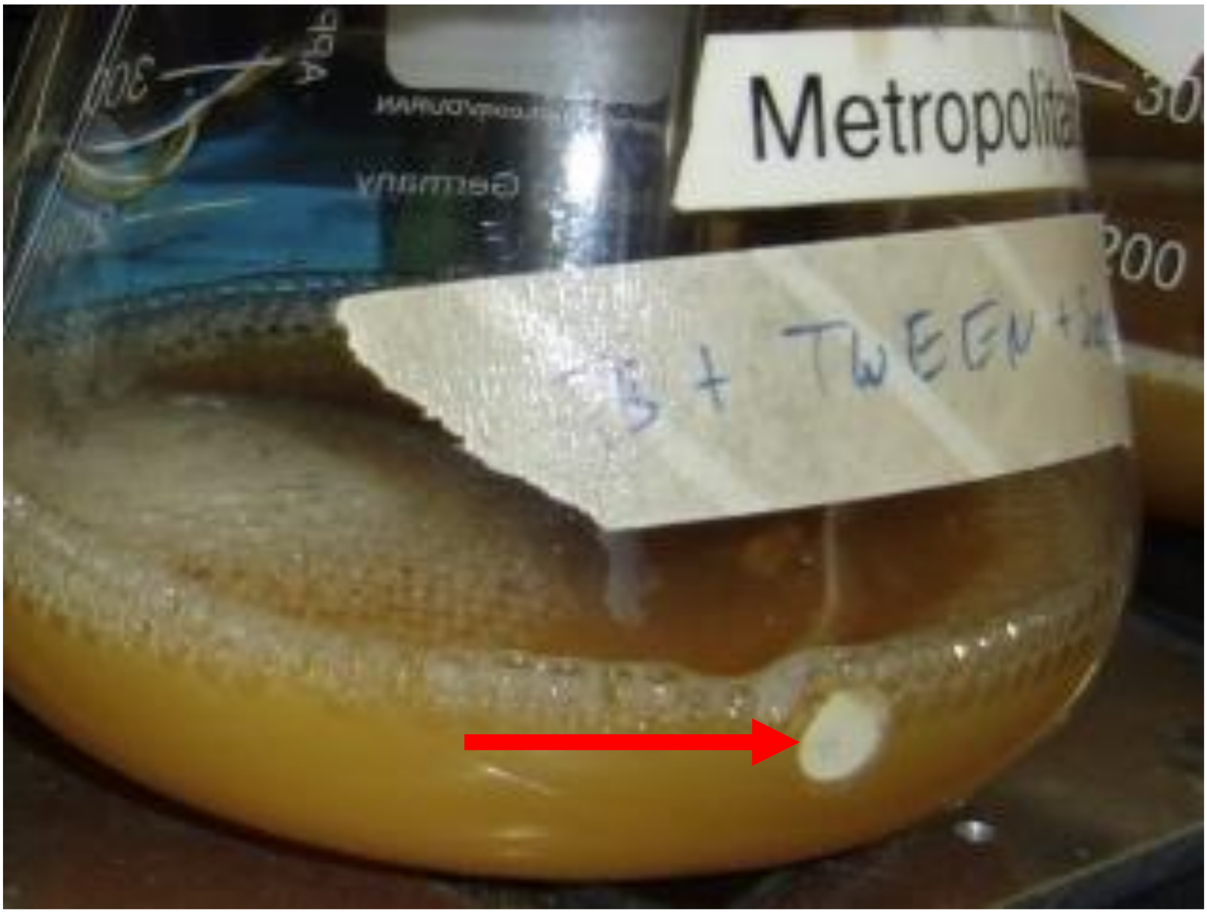

Supplementary Fig. S3 Close-up view of the right shake flask from Supplementary Fig. S2 (B). The sensor spot was glued to the inner glass wall of the shake flask at the height of the largest inner diameter. Picture was taken after a cultivation time of 40 h. Red arrow indicates the position of the sensor spot. Cultivation conditions: TB medium (5 g/L glycerol, 72 mM K_2HPO_4, 17 mM KH_2PO_4, pH_{Start} = 7.5), flask volume (V_F) = 500 mL, filling volume (V_L) = 100 mL, shaking frequency (n) = 250 rpm, shaking diameter (d_0) = 50 mm, temperature (T) = 37 °C.

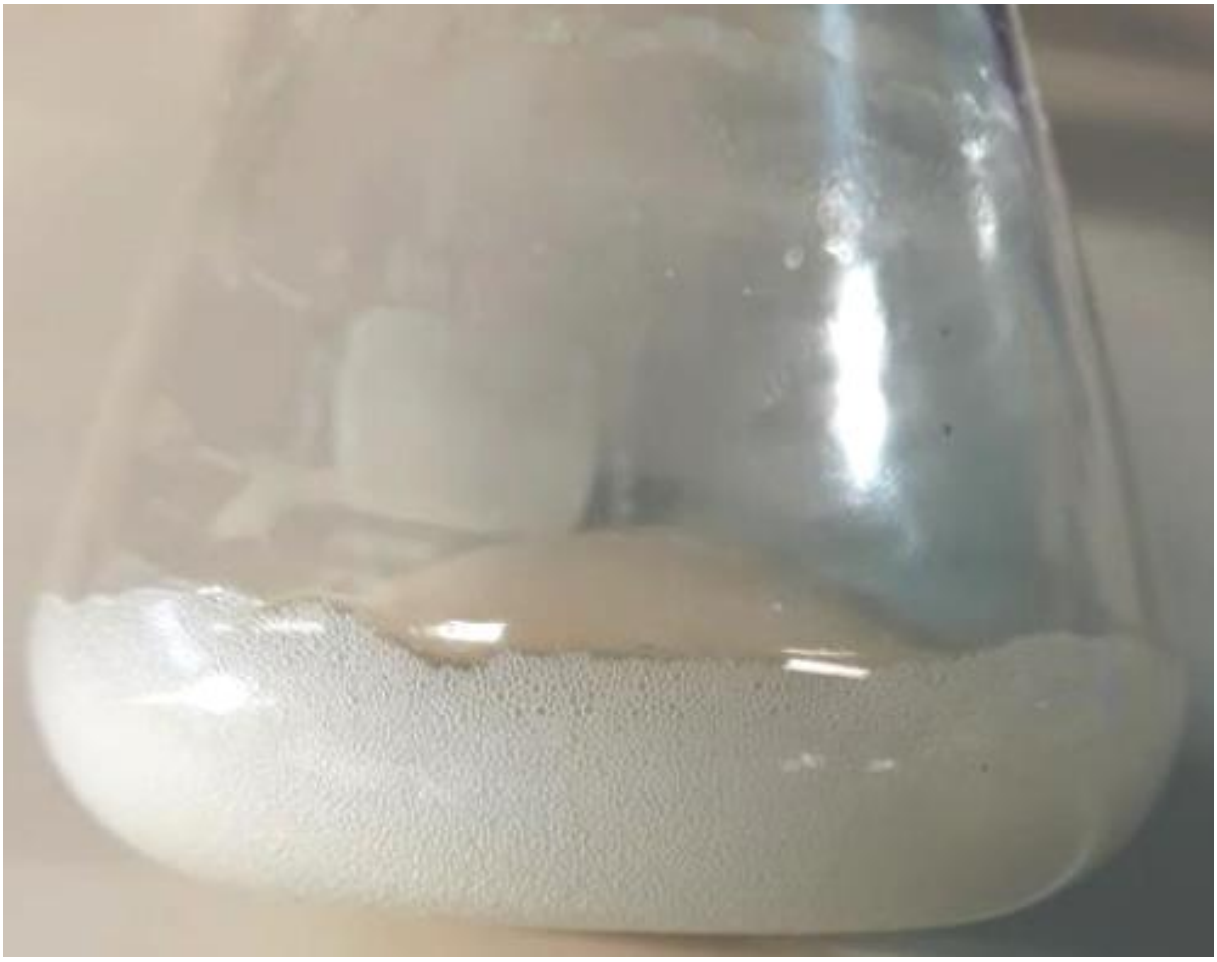

Supplementary Fig. S4 Picture of a foaming cultivation broth of *Bacillus subtilis* P_{xyl} Δspo from Fig. 4 with an initial glucose concentration of 40 g/L. The picture was taken at the beginning of foaming, indicated by the vertical green dashed line in Fig. 4. Cultivation conditions: Modified V3 glucose minimal medium (40 g/L glucose, 0.4 M MOPS buffer, pH_{Start} = 8.1), flask volume (V_F) = 250 mL, filling volume (V_L) = 20 mL, shaking frequency (n) = 350 rpm, shaking diameter (d_0) = 50 mm, temperature (T) = 37 °C. Figure adapted from Hoffmann [38].

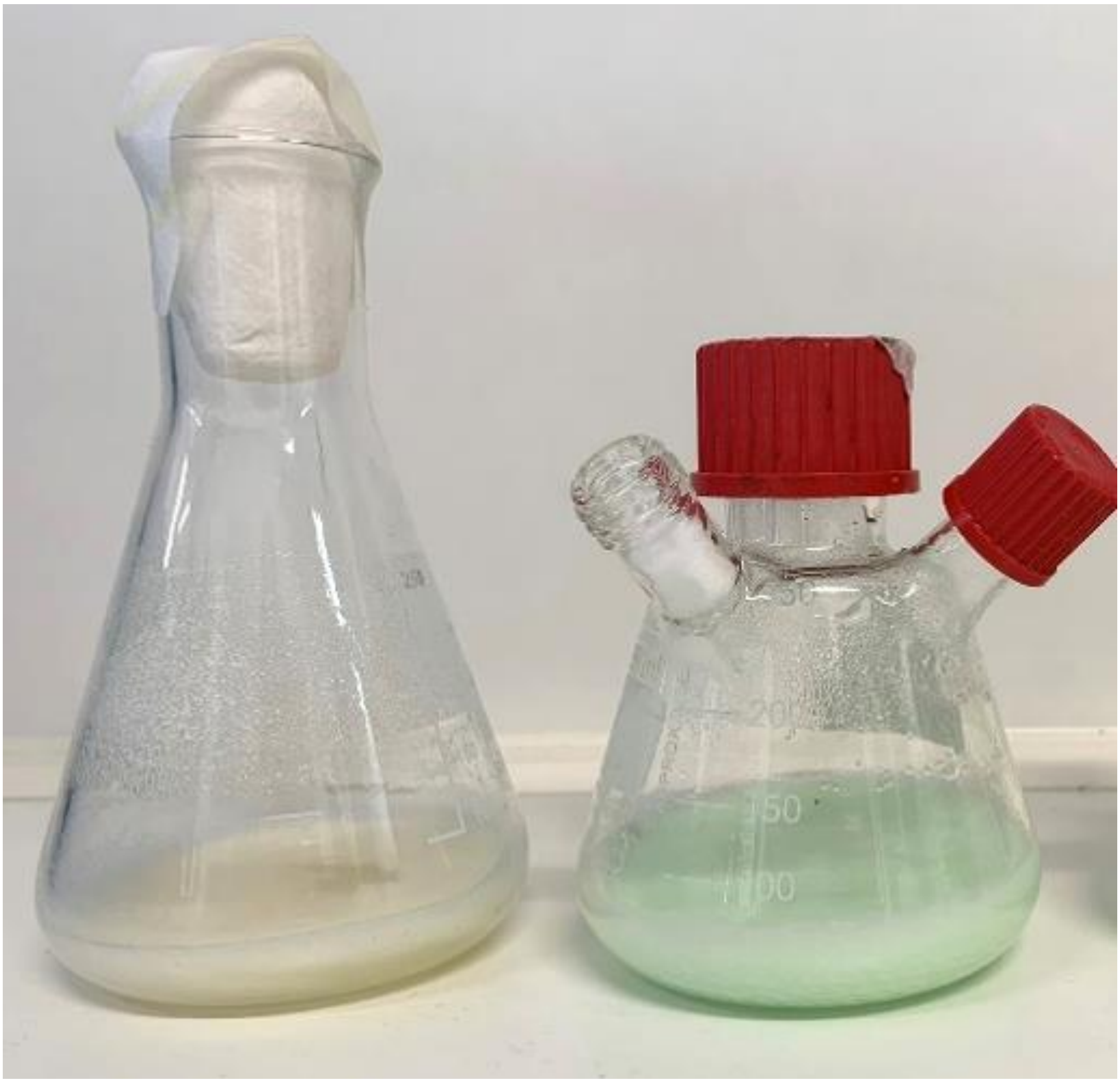

Supplementary Fig. S5 Shake flask and RAMOS flask with foaming cultivation broth at the end of the cultivation of *Paenibacillus polymyxa* (DSM365) from Fig. 5. Green color of the cultivation broth in RAMOS flask caused by fluorescent Oxnano nanoparticles, added to the cultivation broth for online measurements. Cultivation conditions: MM1P100 medium (30 g/L glucose, 12 mM KH_2PO_4, pH_{Start} =7.0), 0.2 mg/mL fluorescent Oxnano nanoparticles (in RAMOS flask), flask volume (V_F) = 250 mL, filling volume (V_L) = 30 mL, shaking frequency (n) = 200 rpm, shaking diameter (d_0) = 50 mm, temperature (T) = 30 °C. Figure adapted from Dinter et al. [19].

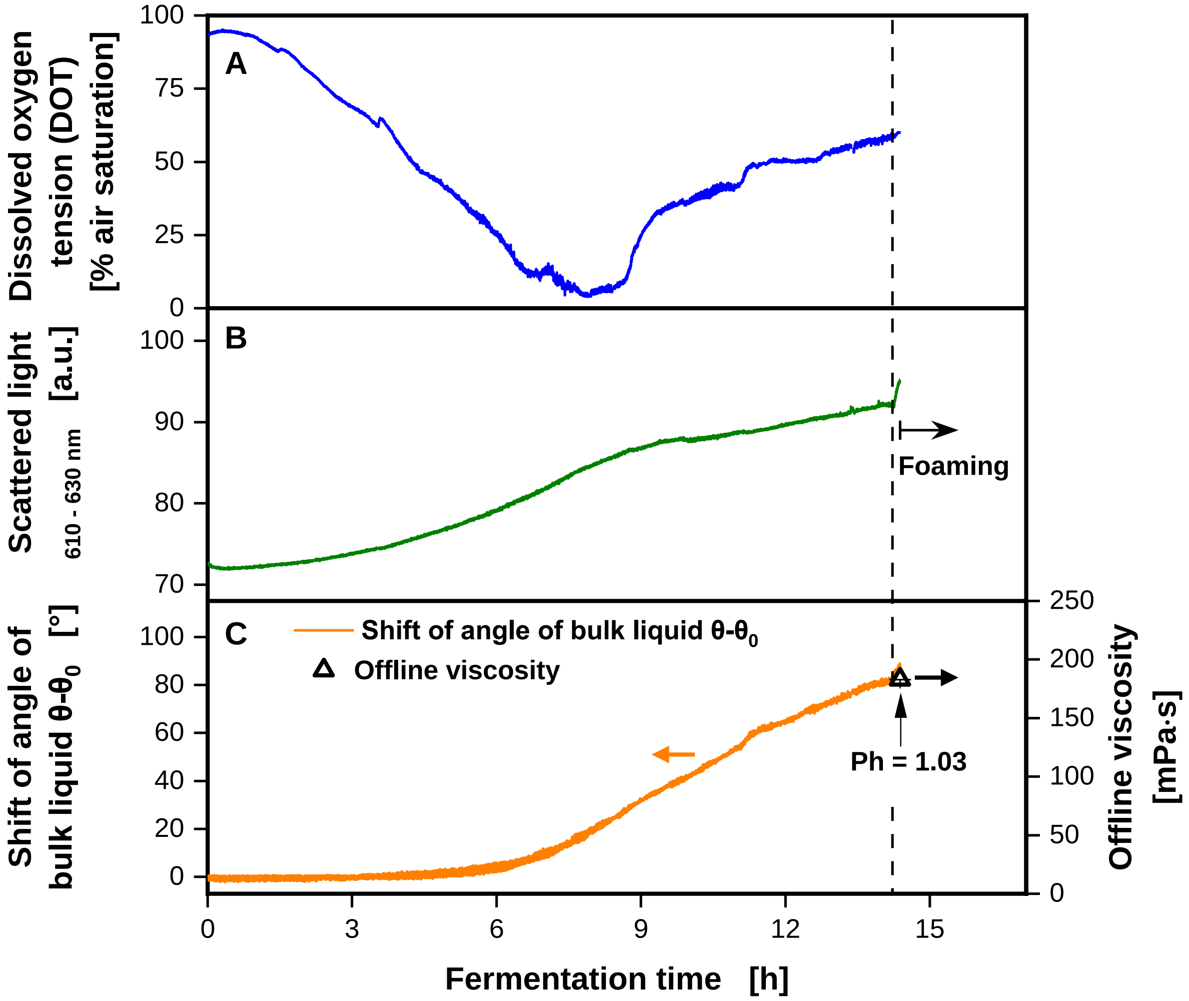

Supplementary Fig. S6 Repetition of the cultivation of *Paenibacillus polymyxa* (DSM365) from Fig. 5. (A) Online monitoring of dissolved oxygen tension. (B) Online monitoring of scattered light. (C) Online monitoring of the shift of angle of the bulk liquid relative to the direction of the centrifugal acceleration $\theta-\theta_0$, compared to the offline viscosity, measured from an offline sample with a rheometer. The phase number (Ph) was calculated from offline measured viscosity. The vertical black dashed line marks the beginning of foaming, indicated by the sudden increase of the scattered light signal. Cultivation conditions: MM1P100 medium (30 g/L glucose, 12 mM KH_2PO_4, pH_{Start} =7), 0.2 mg/mL fluorescent Oxnano nanoparticles, flask volume (V_F) = 250 mL, filling volume (V_L) = 30 mL, shaking frequency (n) = 200 rpm, shaking diameter (d_0) = 50 mm, temperature (T) = 30 °C. Offline viscosity measurements were performed in duplicate.

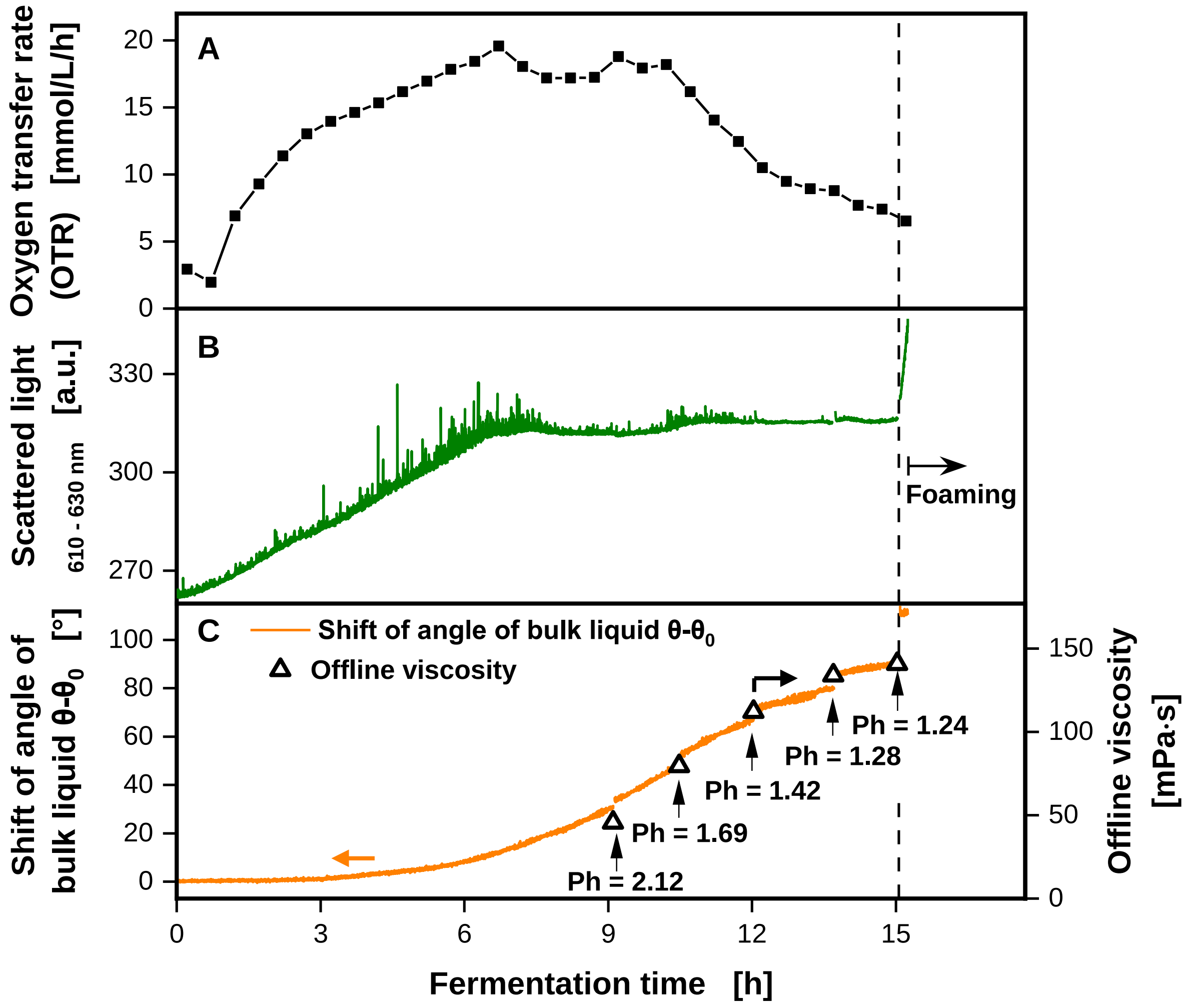

Supplementary Fig. S7 Repetition of the cultivation of *Paenibacillus polymyxa* (DSM365) from Fig. 5 with periodic sampling. (A) Online monitoring of oxygen transfer rate. (B) Online monitoring of scattered light. (C) Online monitoring of the shift of angle of the bulk liquid relative to the direction of the centrifugal acceleration θ-θ_0, compared to the offline viscosity, measured from offline samples with a rheometer. The phase numbers (Ph) were calculated from offline measured viscosity. The vertical black dashed line marks the beginning of foaming, indicated by the sudden increase of the scattered light signal. Cultivation conditions: MM1P100 medium (30 g/L glucose, 12 mM KH_2PO_4, pH_{Start} =7), 0.2 mg/mL fluorescent Oxnano nanoparticles, flask volume (V_F) = 250 mL, filling volume (V_L) = 30 mL, shaking frequency (n) = 200 rpm, shaking diameter (d_0) = 50 mm, temperature (T) = 30 °C. Offline viscosity was measured in single measurement.

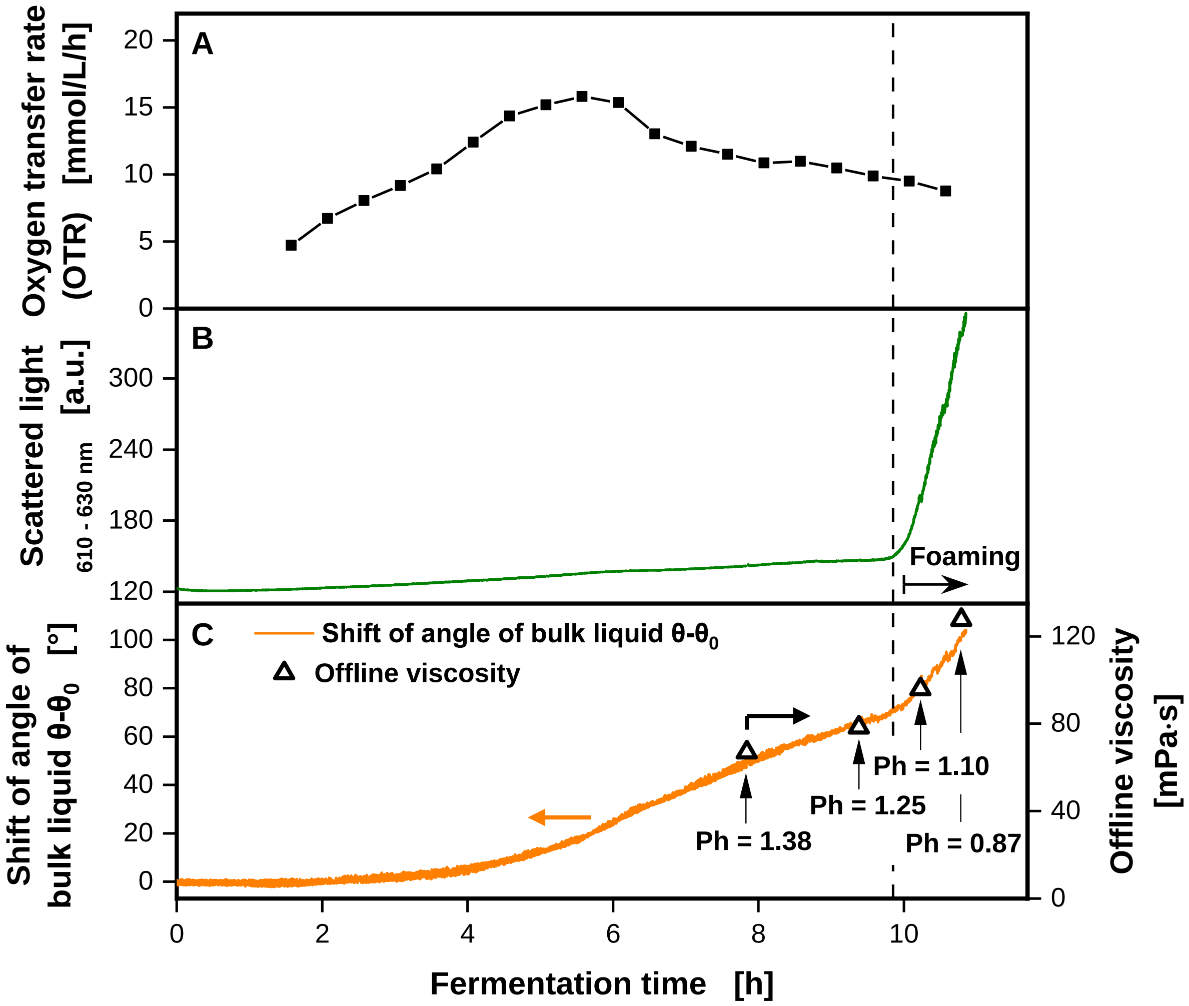

Supplementary Fig. S8 Repetition of the cultivation of *Paenibacillus polymyxa* (DSM365) from Fig. 5 with periodic sampling. (A) Online monitoring of oxygen transfer rate with a RAMOS device. (B) Online monitoring of scattered light. (C) Online monitoring of the shift of angle of the bulk liquid relative to the direction of the centrifugal acceleration θ-θ_0, compared to the offline viscosity, measured from offline samples with a rheometer. The phase numbers (Ph) were calculated from offline measured viscosity. The vertical black dashed line marks the beginning of foaming, indicated by the sudden increase of the scattered light signal. Cultivation conditions: MM1P100 medium (30 g/L glucose, 12 mM KH_2PO_4, pH_{Start} =7), 0.2 mg/mL fluorescent Oxnano nanoparticles, flask volume (V_F) = 250 mL, filling volume (V_L) = 20 mL, shaking frequency (n) = 200 rpm, shaking diameter (d_0) = 50 mm, temperature (T) = 30 °C. Offline viscosity was measured in single measurement.
